# Endoscope-assisted vitrectomy using a 25-gauge system for endophthalmitis: a case series

**DOI:** 10.1186/s12886-026-05068-1

**Published:** 2026-06-24

**Authors:** Mitsuru Otsubo, Shun Konno, Ami Konno, Harumasa Yokota, Taiji Nagaoka

**Affiliations:** Department of Ophthalmology, Asahikawa Medical University, 2-1-1-1 Midorigaoka Higashi, Asahikawa, Hokkaido, 078-8510 Japan

**Keywords:** Endoscope, 25G vitrectomy, Endophthalmitis, Corneal opacity

## Abstract

**Purpose:**

To present a series of seven cases of postoperative endophthalmitis managed with 25-gauge endoscope-assisted vitrectomy using a 10,000-pixel endoscope and to outline the surgical technique and clinical outcomes.

**Methods:**

This study included seven consecutive cases of postoperative endophthalmitis treated with 25-gauge endoscope-assisted vitrectomy at Asahikawa Medical University Hospital between November 2024 and September 2025. Anesthesia was selected based on ocular pain severity: sub-Tenon’s for mild pain and retrobulbar for severe pain. Best-corrected visual acuity (BCVA) before surgery and at the final visit was compared using the Wilcoxon signed-rank test.

**Results:**

Seven cases (5 males and 2 females) were included. The mean age was 74.0 ± 9.1 years. Prior surgeries included cataract surgery (PEA + IOL) in three cases, pars plana vitrectomy in one case, and filtration surgery in three cases. Preoperative visual acuity was light perception in one case and hand motion in six cases. Retrobulbar anesthesia was used in five cases and sub-Tenon’s anesthesia in two cases. Peripheral vitreous shaving was successfully performed without scleral indentation in all cases. Visual acuity improved in all cases, with a mean final BCVA of logMAR 0.36 ± 0.35 (*p* = 0.022).

**Conclusion:**

Endoscope-assisted vitrectomy may be a useful surgical option for postoperative endophthalmitis.

## Introduction

Bacterial endophthalmitis is one of the most serious complications following ophthalmic surgery [1]. The incidence has been reported to be approximately 0.1% after cataract surgery [2].

In recent years, early vitrectomy has been suggested to contribute to improved visual outcomes [3–5], However, corneal edema and opacity often impair intraocular visualization under a surgical microscope, and severe intraocular inflammation can cause significant intraoperative pain [6–8].

Endoscope-assisted vitrectomy has been reported as a useful technique for managing endophthalmitis; however, most previous studies have utilized 20-gauge or 23-gauge systems, and reports using a 25-gauge endoscope remain limited [7, 9–11]. The 25-gauge system is minimally invasive and may be more compatible with vitrectomy under local anesthesia.

We previously reported three cases of bleb-related endophthalmitis treated with 25-gauge endoscope-assisted vitrectomy [12]. However, that report was limited to a disease-specific pilot study. Unlike our previous pilot report limited to bleb-related endophthalmitis, the present study included postoperative endophthalmitis associated with multiple surgical backgrounds, including cataract surgery and pars plana vitrectomy, allowing broader evaluation of the surgical strategy. In the present study, we analyzed seven cases of postoperative endophthalmitis regardless of the type of prior surgery, aiming to establish a practical surgical strategy for safe transition from anterior chamber irrigation to pars plana vitrectomy in eyes with compromised microscopic visualization.

## Methods

This study included seven consecutive cases of postoperative endophthalmitis treated with 25-gauge endoscope-assisted vitrectomy at Asahikawa Medical University Hospital between November 2024 and September 2025. Postoperative endophthalmitis was diagnosed based on anterior chamber inflammation with hypopyon, vitreous opacity on B-scan ultrasonography and rapid visual deterioration following intraocular surgery. Endoscope-assisted vitrectomy was selected based on the anticipated difficulty of microscopic visualization and the expected intraoperative pain associated with scleral indentation under local anesthesia. This was a retrospective consecutive case series. The previously published cases were included in the present analysis [12].

The study was approved by the Institutional Review Board of Asahikawa Medical University (Approval No. 24072) and adhered to the tenets of the Declaration of Helsinki. Written informed consent was obtained from all patients.

Endoscopic guidance was primarily used for intraocular visualization, safe confirmation of infusion cannula placement and peripheral vitreous management when microscopic visualization was compromised by corneal edema, fibrin, or vitreous opacity. The choice of anesthesia was based on the severity of ocular pain: sub-Tenon’s anesthesia was used for mild pain, whereas retrobulbar anesthesia was used for severe pain. All surgeries were performed using the Constellation system (Alcon, Geneva, Switzerland) with a 10,000-pixel 25-gauge endoscope (Fibertech, Tokyo, Japan).

Approximately 0.1–0.2 mL aqueous humor was aspirated using a 1-mL syringe with a 30-gauge needle before anterior chamber irrigation. The samples were immediately submitted for microbiological examination. Anaerobic transport tubes were not used. After aqueous humor sampling, anterior chamber irrigation was performed through a 2.4-mm temporal corneal incision or a previously created cataract incision. A side port (approximately 1 mm) was created at the limbus to establish anterior chamber infusion. Two trocars were inserted at the 2 o’clock and 10 o’clock positions, 3.5–4 mm posterior to the limbus. Anterior chamber irrigation and initial anterior vitrectomy were generally performed under microscopic visualization. Subsequently, an additional trocar was inserted at the 8 o’clock position in the right eye or the 4 o’clock position in the left eye. The infusion cannula was placed and the tip of the cannula was directly visualized within the vitreous cavity using an endoscope before initiating infusion. Peripheral vitrectomy was performed under endoscopic guidance without scleral indentation. Vitreous opacity was removed to the extent that the ora serrata could be visualized endoscopically. Vitreous over the ciliary body was not aggressively dissected but was thinned as much as safely possible.

The irrigation solution contained ceftazidime (40 µg/mL) and vancomycin (20 µg/mL). At the end of surgery, 0.1 mL of ceftazidime (20 mg/mL) and vancomycin (10 mg/mL) were administered intravitreally.

Postoperatively, topical levofloxacin and cefmenoxime were administered every 2 h, and betamethasone eye drops were given six times daily and tapered gradually. Intravenous carbapenem antibiotics were administered in all cases. The duration of administration was approximately one week and was adjusted according to the clinical course in each case.

Best-corrected visual acuity (BCVA) before surgery and at the final visit was compared using the Wilcoxon signed-rank test. Decimal visual acuity was converted to logMAR units. Based on previous reports, hand motion and light perception were assigned logMAR values of 2.3 and 2.7, respectively [13, 14]. Statistical analyses were performed using EZR (Saitama Medical Center, Jichi Medical University, Saitama, Japan), and a p-value < 0.05 was considered statistically significant.

## Results

Seven consecutive cases (5 males and 2 females) were included. The mean age was 74.0 ± 9.1 years. Patient demographics and clinical characteristics are summarized in Table 1. Prior surgeries included cataract surgery (phacoemulsification with intraocular lens implantation [PEA + IOL]) in three cases, pars plana vitrectomy (PPV) in one case, and filtration surgery in three cases. Preoperative visual acuity was light perception in one case and hand motion in six cases, indicating severe visual impairment in all patients.

**Table 1:** Patient demographics and clinical characteristics

Gender (male/female)	5 / 2
Age (years)	74.0 ± 9.1
Prior surgery	
PEA + IOL	3
PPV	1
Filtration surgery	3
Preoperative BCVA	
HM	6
LP	1
Last visit (months)	4.6 ± 2.1
Preoperative BCVA (LogMAR)	2.36 ± 0.15
Postoperative BCVA (LogMAR)	0.36 ± 0.35

Notes: Comparison of preoperative and postoperative BCVA was performed using the Wilcoxon signed-rank test (*p* = 0.022). Abbreviations: BCVA, best-corrected visual acuity; PEA, phacoemulsification; IOL, intraocular lens; PPV, pars plana vitrectomy; HM, hand motion; LP, light perception

The clinical characteristics and surgical details of each patient are summarized in Table 2. The interval from prior surgery to vitrectomy for endophthalmitis ranged from 3 to 5850 days. Endophthalmitis following filtration surgery occurred as delayed-onset infection associated with bleb infection.

**Table 2:** Clinical characteristics, surgical procedures, and outcomes of each patient

Case	Age	Gender	Lateral	Prior surgery	Days from prior surgery to vitrectomy for endophthalmitis	Days from Onset to Surgery	Indication for endoscopic assistance	procedure	Anesthesia	Surgery time (min)	Culture	Preoperative visual acuity	Final logMAR visual acuity	Last visit (month)	Additional intraocular surgery
1	82	Male	R	PEA + IOL	5	2	Corneal edema, poor mydriasis	PPV + IOL removal	Retrobulbar	50	MRSA	LP	0.10	10	IOLSF
2	77	Female	R	PEA + IOL	11	2	Corneal edema	PPV	Sub-Tenon’s	40	Negative	HM	0.00	3	None
3	75	Male	L	PEA + IOL	4	1	Corneal edema with stromal opacity	PPV	Retrobulbar	46	Negative	HM	-0.08	3	None
4	81	Male	R	PPV	3	0	Corneal edema	PPV + SO	Sub-Tenon’s	49	Negative	HM	0.47	3	None
5	58	Female	L	EX-PRESS	956	1	Corneal edema	PPV + EX-PRESS removal + bleb reconstruction	Retrobulbar	100	Negative	HM	0.52	12	AGV
6	65	Male	L	TLE	5850	0	Corneal edema, poor mydriasis	PPV + bleb reconstruction	Retrobulbar	71	Negative	HM	0.82	10	None
7	80	Male	L	TLE	1440	1	Corneal edema, poor mydriasis	PPV + bleb reconstruction	Retrobulbar	48	Negative	HM	0.70	6	None

Abbreviations: PEA, phacoemulsification; IOL, intraocular lens; PPV, pars plana vitrectomy; SO, silicone oil; EX-PRESS, EX-PRESS glaucoma filtration device; TLE, trabeculectomy; MRSA, methicillin-resistant *Staphylococcus aureus*; HM, hand motion; LP, light perception; IOLSF, intraocular lens scleral fixation; AGV, Ahmed glaucoma valve

All patients underwent 25-gauge endoscope-assisted vitrectomy. Surgical procedures included PPV alone (2 cases), PPV with IOL removal (1 case), PPV with silicone oil tamponade (1 case), PPV with EX-PRESS removal and bleb reconstruction (1 case), and PPV with bleb reconstruction (2 cases). Case 4 developed postoperative endophthalmitis after pars plana vitrectomy for rhegmatogenous retinal detachment. No recurrent retinal detachment or iatrogenic retinal breaks were identified during surgery. Silicone oil tamponade was performed because residual vitreous contraction and the potential risk of recurrent retinal detachment were considered. Retrobulbar anesthesia was used in five cases and sub-Tenon’s anesthesia in two cases.

The surgical time ranged from 40 to 100 min. Procedures for post-filtration endophthalmitis tended to be longer due to the need for bleb manipulation. Peripheral vitreous shaving was successfully performed without scleral indentation in all cases. Culture results were positive for methicillin-resistant *Staphylococcus aureus* (MRSA) in one case and negative in six cases.

Visual acuity improved in all cases, with a mean final BCVA of logMAR 0.36 ± 0.35 (Wilcoxon signed-rank test, *p* = 0.022). Additional intraocular surgery was required in two cases (IOL scleral fixation in one case and Ahmed glaucoma valve implantation in one case). No iatrogenic retinal breaks occurred intraoperatively. No postoperative retinal detachment, persistent hypotony, or recurrent endophthalmitis developed during follow-up.

### Representative case 1

An 82-year-old male underwent PEA + IOL in the right eye at a local clinic. On postoperative day 3, blurred vision and ocular pain developed. On postoperative day 5, due to lack of improvement, he was referred to our hospital with suspected postoperative endophthalmitis.

**Fig. 1:**
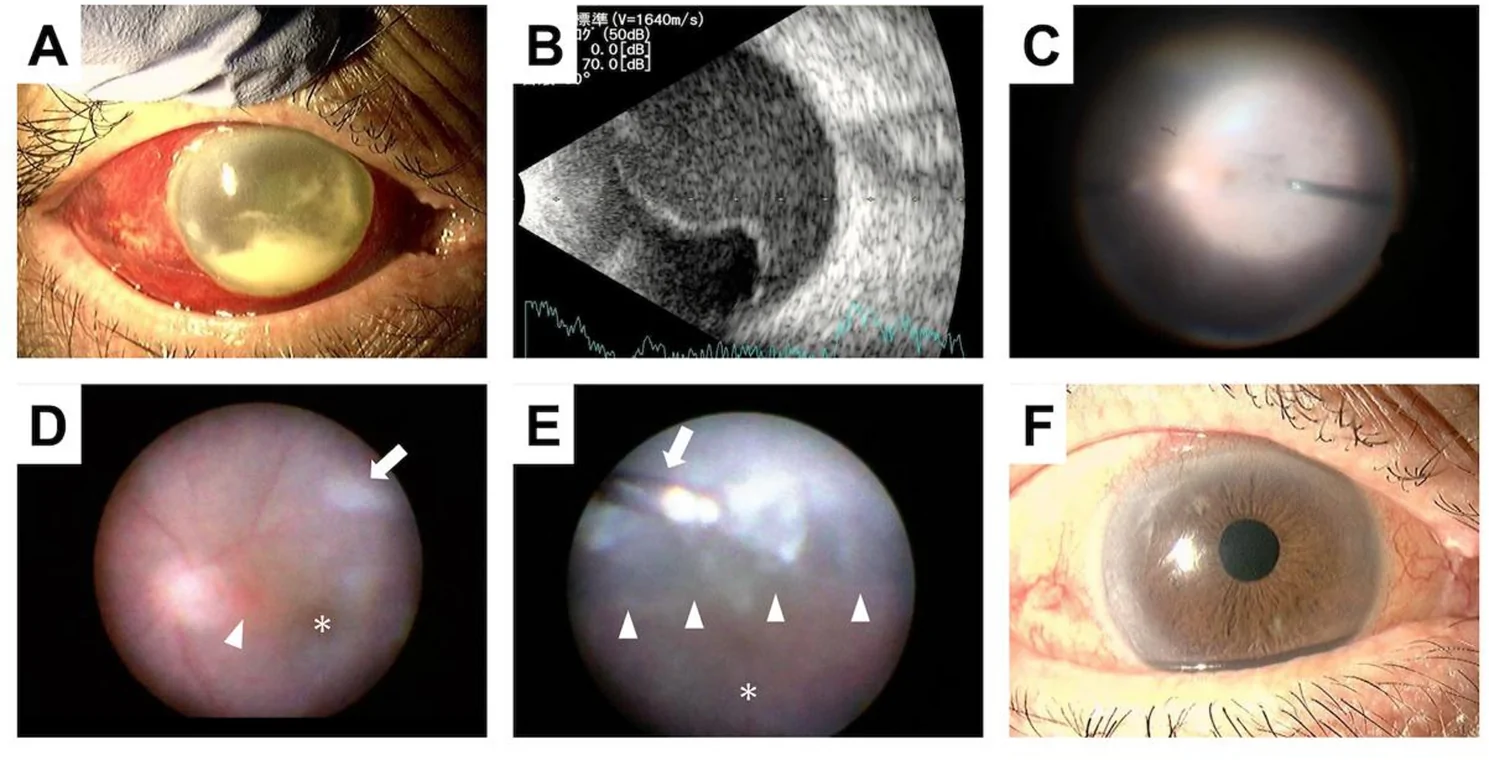
Representative case of postoperative endophthalmitis treated with 25-gauge endoscope-assisted vitrectomy. (**A**) Anterior segment photograph showing corneal edema and dense hypopyon, with no fundus visibility. (**B**) B-scan ultrasonography demonstrating dense vitreous opacities. (**C**) Intraoperative microscopic view after removal of hypopyon, with limited visualization due to media opacity. (**D**) Intraoperative endoscopic view showing improved visualization of intraocular structures, enabling safe vitrectomy. White arrows indicate retinal white lesions, white arrowheads indicate retinal hemorrhages, and the asterisk indicates the fovea. (**E**) Intraoperative peripheral endoscopic view during peripheral vitrectomy under endoscopic guidance. White arrows indicate the vitreous cutter, white arrowheads indicate the ora serrata, and the asterisk indicates the peripheral retina. (**F**) Postoperative fundus photograph demonstrating resolution of inflammation

At presentation, visual acuity was light perception, and intraocular pressure was 6 mmHg. Corneal edema and dense hypopyon were observed, and fundus visualization was not possible (Fig. 1A). B-scan ultrasonography revealed vitreous opacities (Fig. 1B).

A diagnosis of postoperative endophthalmitis was made, and 25-gauge endoscope-assisted vitrectomy with IOL removal was performed on the same day. Representative intraoperative findings under microscopic and endoscopic views are shown in Fig. 1C, D and E. Due to the high activity of infection, the IOL was removed for infection control.

Postoperatively, culture of intraocular samples revealed MRSA. The infection subsequently resolved (Fig. 1F). Further examination revealed nasolacrimal duct obstruction and chronic dacryocystitis. Dacryocystorhinostomy was performed 7 months after vitrectomy. Following resolution of dacryocystitis, scleral fixation of the IOL was performed, and the final BCVA improved to logMAR 0.10.

## Discussion

In this case series, all seven patients with postoperative endophthalmitis underwent successful vitrectomy under local anesthesia, despite severe preoperative visual impairment, with a mean final BCVA of logMAR 0.36 ± 0.35.

Vitrectomy for endophthalmitis is often complicated by poor intraocular visualization due to corneal edema and opacity, as well as intraoperative pain associated with severe inflammation [6–8]. Endoscopic visualization enables intraocular observation independent of anterior segment opacity and allows visualization of the peripheral vitreous and ciliary body without scleral indentation. These characteristics likely contributed to the successful completion of all surgeries under local anesthesia in this series.

In our surgical approach, anterior vitrectomy was first performed under anterior chamber infusion through a corneal side port, followed by insertion of the infusion cannula via the pars plana. The tip of the infusion cannula was directly visualized within the vitreous cavity using an endoscope before initiating infusion. Although this method ensures safe infusion placement, caution is required in eyes with zonular weakness, as it may increase the risk of IOL instability or dislocation.

In conventional microscopic vitrectomy for endophthalmitis, safe visualization of the peripheral vitreous and confirmation of infusion cannula placement may be challenging in eyes with severe anterior segment inflammation or dense vitreous opacity. In the present series, endoscopic guidance enabled direct confirmation of pars plana infusion cannula placement and peripheral vitreous management without scleral indentation, which may be particularly advantageous in inflamed eyes undergoing surgery under local anesthesia.

Previous reports of endoscope-assisted vitrectomy for endophthalmitis have primarily focused on visualization through opaque media [7, 9–12]. In contrast, the present study additionally emphasized a practical surgical strategy for safe transition from anterior chamber irrigation to pars plana vitrectomy under compromised visualization. In bleb-related endophthalmitis, avoidance of scleral indentation may contribute to preservation of the conjunctiva and filtering bleb. In the newly included cases of acute postoperative endophthalmitis after cataract surgery or pars plana vitrectomy, endoscopic guidance was considered useful mainly for safe intraocular visualization and peripheral vitreous management when microscopic visualization was insufficient because of corneal edema, fibrin, hypopyon, or dense vitreous opacity. In acute postoperative endophthalmitis particularly after cataract surgery, avoidance of scleral indentation may also help minimize surgical stress on recent corneal wounds. Although Dib et al. advocated more extensive vitrectomy compared with the original Endophthalmitis Vitrectomy Study paradigm, the necessity of extensive peripheral vitreous shaving in endophthalmitis surgery remains debatable [1, 3]. Therefore, the present study does not intend to overemphasize peripheral vitreous shaving itself, but rather the practical role of endoscopic guidance in safe peripheral vitreous management under compromised visualization.

In the Endophthalmitis Vitrectomy Study, 33% of patients with postoperative endophthalmitis after cataract surgery achieved a final visual acuity of 20/40 or better following vitrectomy [1]. Although the present series was too small for direct comparison with the Endophthalmitis Vitrectomy Study, all three cases of post-cataract surgery endophthalmitis achieved a final visual acuity of 20/40 or better.

Previous reports of endoscope-assisted vitrectomy for endophthalmitis by De Smet et al. and Dave et al. reported visual improvement to 20/40 or better in 26.7% and 20/400 or better in 15.15% of cases, respectively [7, 9]. However, these studies included more severe cases, such as endophthalmitis associated with open-globe injury or advanced infectious keratitis, making direct comparison difficult.

Previous reports of endoscope-assisted vitrectomy for endophthalmitis have mainly used 20-gauge or larger, as well as 23-gauge systems, whereas reports using 25-gauge endoscopes remain limited [7, 9–11]. In the present study, a 10,000-pixel 25-gauge endoscope provided sufficient intraocular visualization for safe surgical procedures under compromised visualization. However, the present study was not designed to compare image quality with previous endoscopic systems or conventional microscopic observation. Furthermore, the small-gauge system may reduce conjunctival and scleral damage, facilitating wound closure.

In cases of endophthalmitis following filtration surgery, additional glaucoma surgery may be required after infection control. Minimizing conjunctival and scleral damage may therefore be clinically advantageous. In this series, one case required Ahmed glaucoma valve implantation, supporting the potential benefit of a minimally invasive approach.

This study includes three previously reported cases of bleb-related endophthalmitis. However, unlike the previous pilot report, which focused exclusively on bleb-related cases, the present study analyzed postoperative endophthalmitis regardless of the type of prior surgery, thereby further evaluating the role of endoscope-assisted vitrectomy in a broader clinical context.

This study has several limitations, including its retrospective design, small sample size, and lack of a control group. The low culture positivity rate may have been related to prior antibiotic administration before referral and the use of aqueous humor samples alone for microbiological examination. The heterogeneous surgical backgrounds and inflammatory etiologies in the present cohort may have influenced the interpretation of surgical outcomes. However, inclusion of different postoperative conditions allowed evaluation of the practical applicability of the proposed surgical strategy across broader clinical scenarios. Further studies with larger cohorts and comparative analyses with conventional microscopic vitrectomy are warranted.

## Conclusion

Endoscope-assisted vitrectomy may be a useful surgical option for postoperative endophthalmitis, particularly in cases with poor visualization due to corneal opacity, allowing safe procedures under local anesthesia without the need for scleral indentation.

## Data Availability

The datasets used and/or analyzed during the current study are available from the corresponding author on reasonable request.

## Abbreviations

BCVA: Best-corrected visual acuity
PEA + IOL: Phacoemulsification with intraocular lens implantation
PPV: Pars plana vitrectomy
MRSA: Methicillin-resistant Staphylococcus aureus
